# Effects of weather-related social distancing on city-scale transmission of respiratory viruses: a retrospective cohort study

**DOI:** 10.1186/s12879-021-06028-4

**Published:** 2021-04-09

**Authors:** Michael L. Jackson, Gregory R. Hart, Denise J. McCulloch, Amanda Adler, Elisabeth Brandstetter, Kairsten Fay, Peter Han, Kirsten Lacombe, Jover Lee, Thomas R. Sibley, Deborah A. Nickerson, Mark J. Rieder, Lea Starita, Janet A. Englund, Trevor Bedford, Helen Chu, Michael Famulare, on behalf of the Seattle Flu Study Investigators, Helen Y. Chu, Michael Boeckh, Janet A. Englund, Michael Famulare, Barry R. Lutz, Deborah A. Nickerson, Mark J. Rieder, Lea M. Starita, Matthew Thompson, Jay Shendure, Trevor Bedford, Amanda Adler, Elisabeth Brandstetter, Jeris Bosua, Shari Cho, Chris D. Frazar, Peter D. Han, James Hadfield, Shichu Huang, Michael L. Jackson, Anahita Kiavand, Louise E. Kimball, Kirsten Lacombe, Jennifer Logue, Victoria Lyon, Kira L. Newman, Matthew Richardson, Thomas R. Sibley, Monica L. Zigman Suchsland, Caitlin Wolf

**Affiliations:** 1 Kaiser Permanente Washington Health Research Institute, Seattle, WA USA; 2 Institute for Disease Modeling, Bellevue, WA USA; 3 Department of Medicine, University of Washington School of Medicine, Seattle, WA USA; 4 Seattle Children’s Research Institute, Seattle, WA USA; 5 Vaccine and Infectious Disease Division, Fred Hutchinson Cancer Research Center, Seattle, WA USA; 6 Brotman Baty Institute for Precision Medicine, Seattle, WA USA; 7 Department of Genome Sciences, University of Washington, Seattle, WA USA

**Keywords:** Influenza, human, Epidemiology, Respiratory syncytial virus, human, Non-Pharmaceutical Interventions

## Abstract

**Background:**

Unusually high snowfall in western Washington State in February 2019 led to widespread school and workplace closures. We assessed the impact of social distancing caused by this extreme weather event on the transmission of respiratory viruses.

**Methods:**

Residual specimens from patients evaluated for acute respiratory illness at hospitals in the Seattle metropolitan area were screened for a panel of respiratory viruses. Transmission models were fit to each virus to estimate the magnitude reduction in transmission due to weather-related disruptions. Changes in contact rates and care-seeking were informed by data on local traffic volumes and hospital visits.

**Results:**

Disruption in contact patterns reduced effective contact rates during the intervention period by 16 to 95%, and cumulative disease incidence through the remainder of the season by 3 to 9%. Incidence reductions were greatest for viruses that were peaking when the disruption occurred and least for viruses in an early epidemic phase.

**Conclusion:**

High-intensity, short-duration social distancing measures may substantially reduce total incidence in a respiratory virus epidemic if implemented near the epidemic peak. For SARS-CoV-2, this suggests that, even when SARS-CoV-2 spread is out of control, implementing short-term disruptions can prevent COVID-19 deaths.

**Supplementary Information:**

The online version contains supplementary material available at 10.1186/s12879-021-06028-4.

## Main text

Novel respiratory viruses emerge periodically to cause global pandemics [1–3]. During the early stages of a pandemic, community mitigation strategies such as social distancing are among the few effective interventions for reducing transmission and infection. The potential impact and optimal timing of city-wide social distancing interventions to reduce the spread of influenza and other respiratory viruses are largely unknown. Most estimates of social distancing impact are limited to studies of school closures, including both routine holiday closures and reactive closures due to influenza epidemics. School closures may reduce rates of medically attended influenza in school-aged children, although with highly heterogeneous effects (2 to 29% reductions), and with lesser effects on younger children and adults [4–8].

Generalizing to broader social distancing efforts from these studies is difficult, however, as school closures tend to have limited impacts on working-age and older adults, and school-aged children may recongregate outside of schools [8–11]. In February 2019, unusually high snowfall in western Washington State led to widespread school and workplace closures and to reduced regional travel [12]. This disruption of work and travel can be considered a proxy for social distancing that might accompany community-wide social mobility restrictions in the event of a pandemic. The objective of this study was to estimate the impact of this weather-created social distancing on transmission of respiratory viruses in the greater Seattle metropolitan area.

### Methods

The Seattle Flu Study, initiated during the 2018/19 influenza season, is a regional surveillance project which aims to evaluate the transmission of influenza and other respiratory pathogens at a city-wide scale [13]. The Seattle Flu Study had several surveillance arms; this analysis focuses on data from collection and molecular testing of residual specimens from patients evaluated at regional hospitals for acute respiratory illness, as that provided 86% of samples (9199 of 10,696 detected pathogens, from 7555 unique patients; Supplemental Table) and with the broadest regional population representation.

Nucleic acids extracted from respiratory swab specimens from study participants were screened for the presence of multiple respiratory pathogens by reverse-transcription and Taqman. Screened pathogens included influenza A/H1N1, A/H3N2, and B; respiratory syncytial virus (RSV) A and B; human coronavirus (229E, NL63, OC43, HKU1) (hCoV); human metapneumovirus (hMPV); rhinovirus (HRV), and adenovirus (AdV).

Unusually heavy snowfall occurred in the greater Seattle metropolitan area between Feb 3 and Feb 112,019, totaling 20.2 in. at Seattle-Tacoma International Airport over this time. The majority of public schools in the region were closed for at least 5 days, with a weekend in between. City of Seattle public preschools were closed from Feb 9 through Feb 13, and King County public transportation was on emergency service from Feb 9 through Feb 12. We used Washington State Department of Transportation traffic data on regional interstate highways to quantify mobility during January–March 2019 [12], and visits to regional hospitals (Harborview Medical Center, University of Washington Medical Center, and Northwest Hospital) to quantify visit volumes during this period.

We modeled daily counts of Seattle Flu Study specimens testing positive for each of nine respiratory viruses using the Susceptible-Exposed-Infectious-Recovered (SEIR) framework, allowing for decreases both in virus transmission and in probability of detection by surveillance during the period of school and traffic disruption (Fig. 2).

**Fig. 1:**
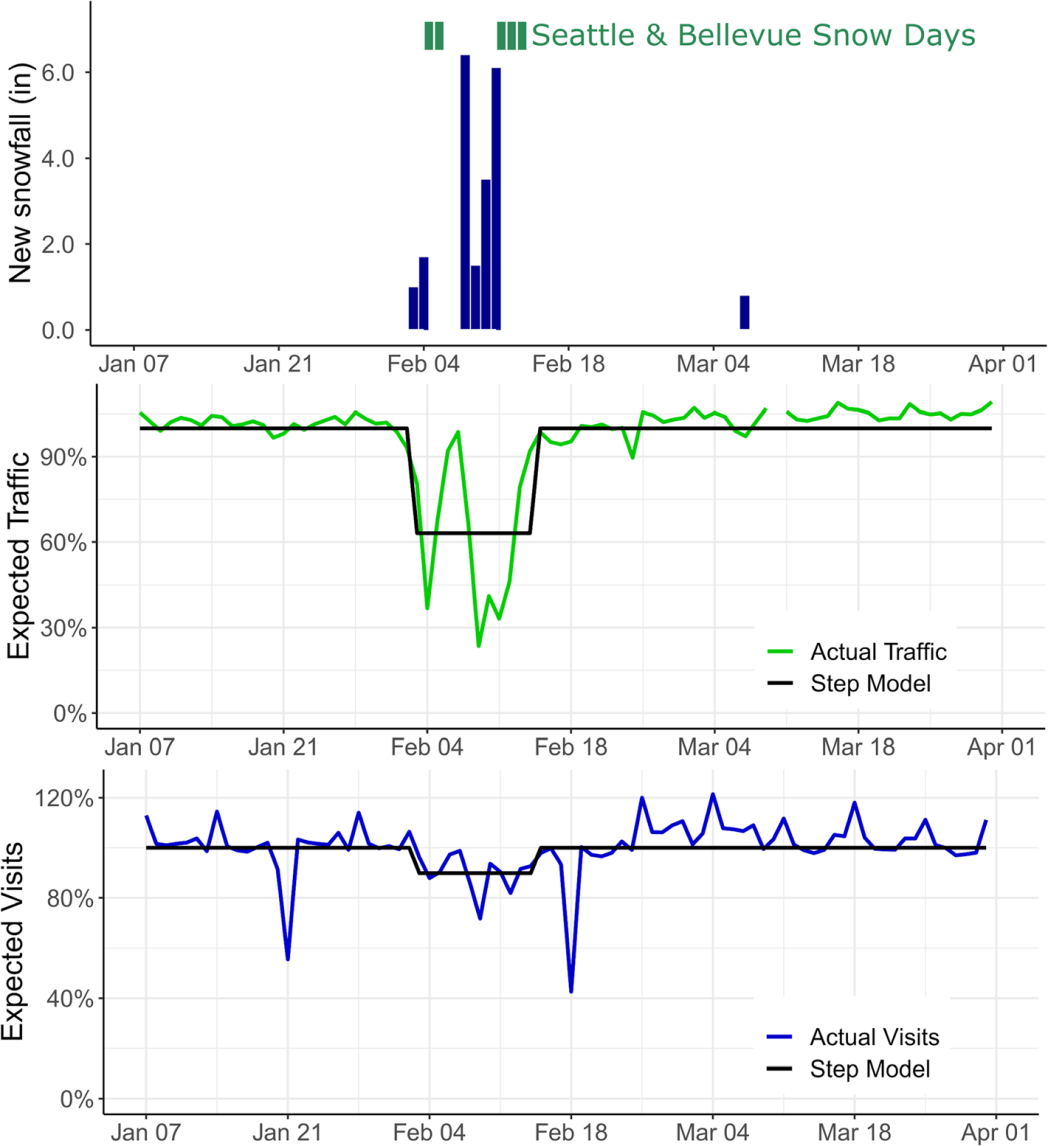
Snowfall, and impact on schools and regional transportation – greater Seattle metropolitan area, Jan–Apr 2019. Top panel: public school closures in two representative districts and snowfall inches at Seattle-Tacoma International Airport; center panel, traffic on regional interstate highways vs. expected (green), with mean disruption due to weather (black); bottom panel: regional daily hospital visitsvs. Expected (blue), with mean disruption due to weather (black)

We modeled each pathogen with a deterministic SEIR transmission model coupled to a simple observation model:

$$ dS/ dt=-\beta \left(1+{\beta}^{\prime }{\theta}_t\right) SI $$

$$ dE/ dt=\beta \left(1+{\beta}^{\prime }{\theta}_t\right) SI-\sigma E $$

$$ dI/ dt=\sigma E-\gamma I $$

$$ dH/ dt=p\left(1+\alpha {\theta}_t\right)\left( dI/ dt\right)+ p\alpha \left(\delta {t}_{start}-{\delta t}_{end}\right)I $$

where *S*, *E*, and *I* are the proportion of the population that is susceptible, infected but not yet infectious, and infectious, respectively; *β* is the effective contact rate, *σ* is the rate of movement from pre-infectious to infectious, and *γ* is the rate of loss of infectiousness. The *β’* parameter is the strength of the interruption to disease transmission during the extreme weather event, which operates on a square pulse *θ*_t_ between times t_start_ and t_end_. We set t_start_ and t_end_ to the 3 Feb and 15 Feb respectively, capturing all the days of snow, school closure, reduced traffic and hospital visits, making the period 12 days long. In the observation model, *H* is the number of observed infections and *p* combines the sampling probability and population size. The *α* parameter is the strength of the interruption of observation during the extreme weather event, which is assumed to be − 10% based on hospital visit data (Fig. 1).

Values for *σ*^*− 1*^ and *γ*^*− 1*^ were estimated from the existing literature for the nine viruses (Table 1) [14–27]. Estimates for *β*, *β*^’^, *p*, and the proportion of the population infected at the start of the season were obtained via maximum likelihood estimation assuming daily observations were a Poisson sample from the underlying prevalence. We calculated confidence intervals by randomly drawing 200 parameter sets from the posterior distribution and generating 100 realizations (with the Poisson noise) of each, removing trajectories below the 2.5 percentile and above the 97.5 percentile. To assess the sensitivity of our findings to the values of the fixed parameters, we re-estimated values for *β*, *β*^’^, *p*, and the initial population infected under different assumed values of the fixed parameters and calculated the percent of infections averted under each assumption (Supplemental Appendix).

**Table 1:** Pathogen-specific parameters and estimated impacts of weather-related contact disruption

Pathogen	Duration of pre-infectious period (days)^a^	Duration of infectious-ness (days)^b^	Number positive samples	Median age of infected subjects (years)	Percent of infected subjects 18 years or older	Estimated basic reproductive number (*R*_0_)	Estimated reduction in effective contact rate	Estimated proportion of infections averted
Influenza A/H1N1	2	5	1413	9.0	47%	1.60 (1.56–64)	27.3% (19.2–35.4%)	7.6% (5.2–9.7%)
Influenza A/H3N2	2	5	1704	9.0	31%	2.18 (2.12–2.28)	66.5% (62.2–74.5%)	3.1% (2.5–3.2%)
Influenza B	2	6	82	7.0	28%	1.78 (1.48–2.09)	83.8% (39.4–100%)	7.8% (5.5–8.1%)
RSV A	5	11	745	2.0	20%	2.33 (2.33–2.47)	94.6% (70.7–100%)	8.8% (6.4–9.1%)
RSV B	5	11	542	2.0	38%	2.03 (1.93–2.17)	85.6% (54.5–100%)	9.2% (6.2–10.3%)
hCoV	3	3.5	1258	6.0	46%	1.38 (1.35–1.41)	16.2% (7.1–24.2%)	5.6% (4.1–6.9%)
AdV	6	5.5	770	3.0	18%	1.72 (1.61–1.83)	49.5% (30.3–65.7%)	6.2% (4.6–7.4%)
HRV	2	11	1077	4.0	35%	1.40 (1.33–1.46)	49.5% (30.3–65.7%)	6.8% (4.7–8.2%)
hMPV	4	10.5	599	4.0	43%	1.99 (1.79–2.19)	52.5% (18.2–79.8%)	3.0% (2.0–3.7%)

^a^Duration of the pre-infectious period (i.e. 1/σ) is the average number of days an individual is in the Exposed (infected but not yet infectious) category in the Susceptible-Exposed-Infectious-Recovered model

^b^Duration of infectiousness (i.e. 1/γ) is the average number of days an individual is in the Infectious category in the Susceptible-Exposed-Infectious-Recovered model

### Results

**Fig. 2:**
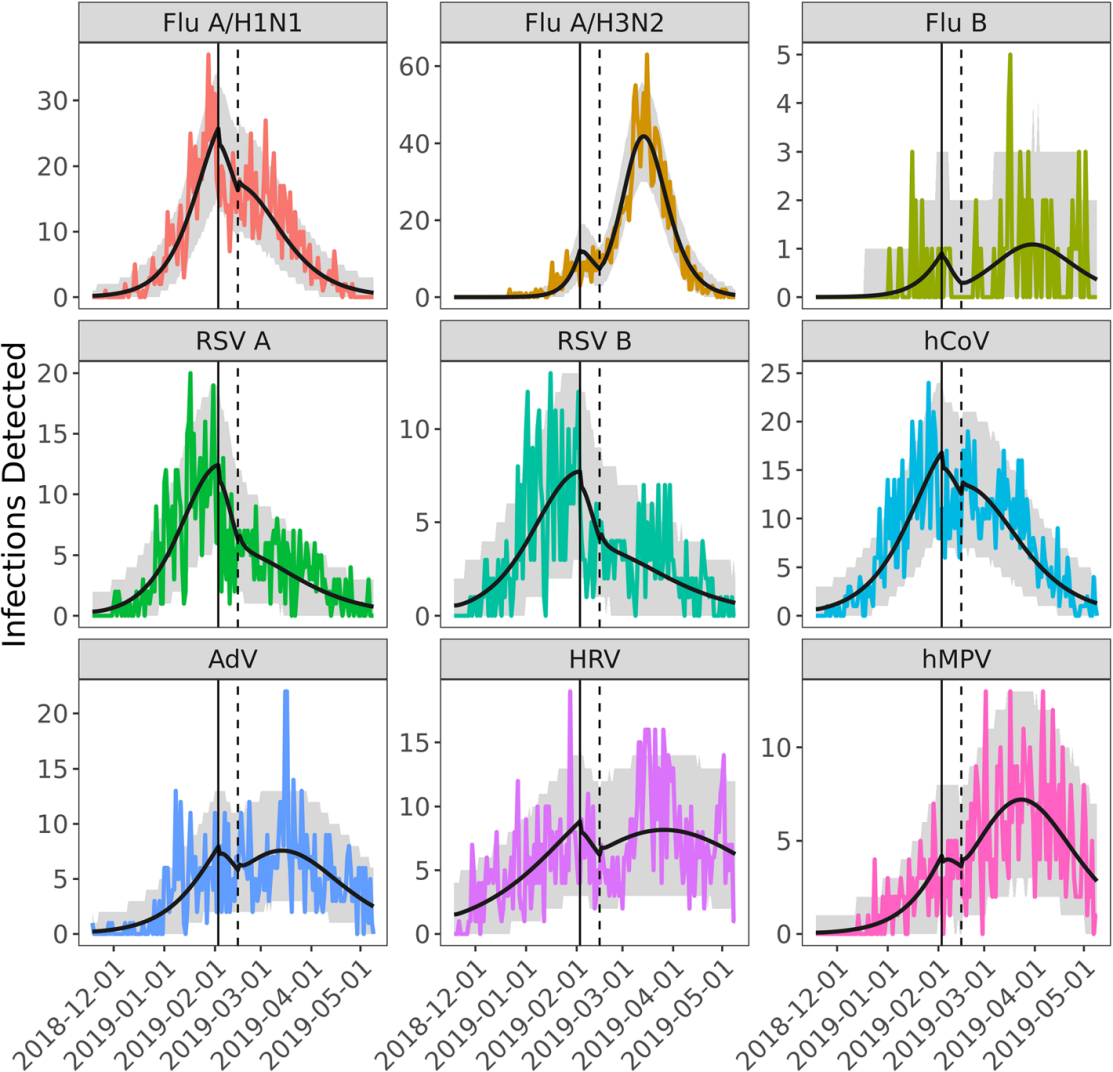
Observed and modeled daily counts of positive laboratory tests for nine respiratory viruses – Nov 2018 to May 2019, greater Seattle metropolitan area. The colored lines show the observed daily incidence for each of the nine pathogens. The black line is the model prediction using the maximum likelihood estimate for model parameters. The gray shading encapsulates the effect of uncertainty in the model parameters (95% CI). The vertical solid and dashed black lines mark the beginning and end of weather-related disruptions

Traffic volumes on major interstate highways in the greater Seattle area ranged between 24 and 90% (mean, 63%) of average daily volume during the 12 day period from Feb 3 to Feb 15, 2019 (Fig. 1). Visits to regional hospitals ranged from 72 to 99% (mean, 90%) of average daily volume over this same period. After fitting SEIR models to each pathogen (Fig. 2), the transient weather-related decrease in effective contact rates ranged from 16.2% (95% CI, 7.1–24.2%) for coronavirus to 94.6% (95% CI, 70.7–100%) for RSV A (Table 1). Decreases in effective contact rates were negatively correlated with the fraction of subjects aged 18 years or older for each pathogen (*R*^2^ = − 0.65, *p* = 0.06), although not significantly so.

**Fig. 3:**
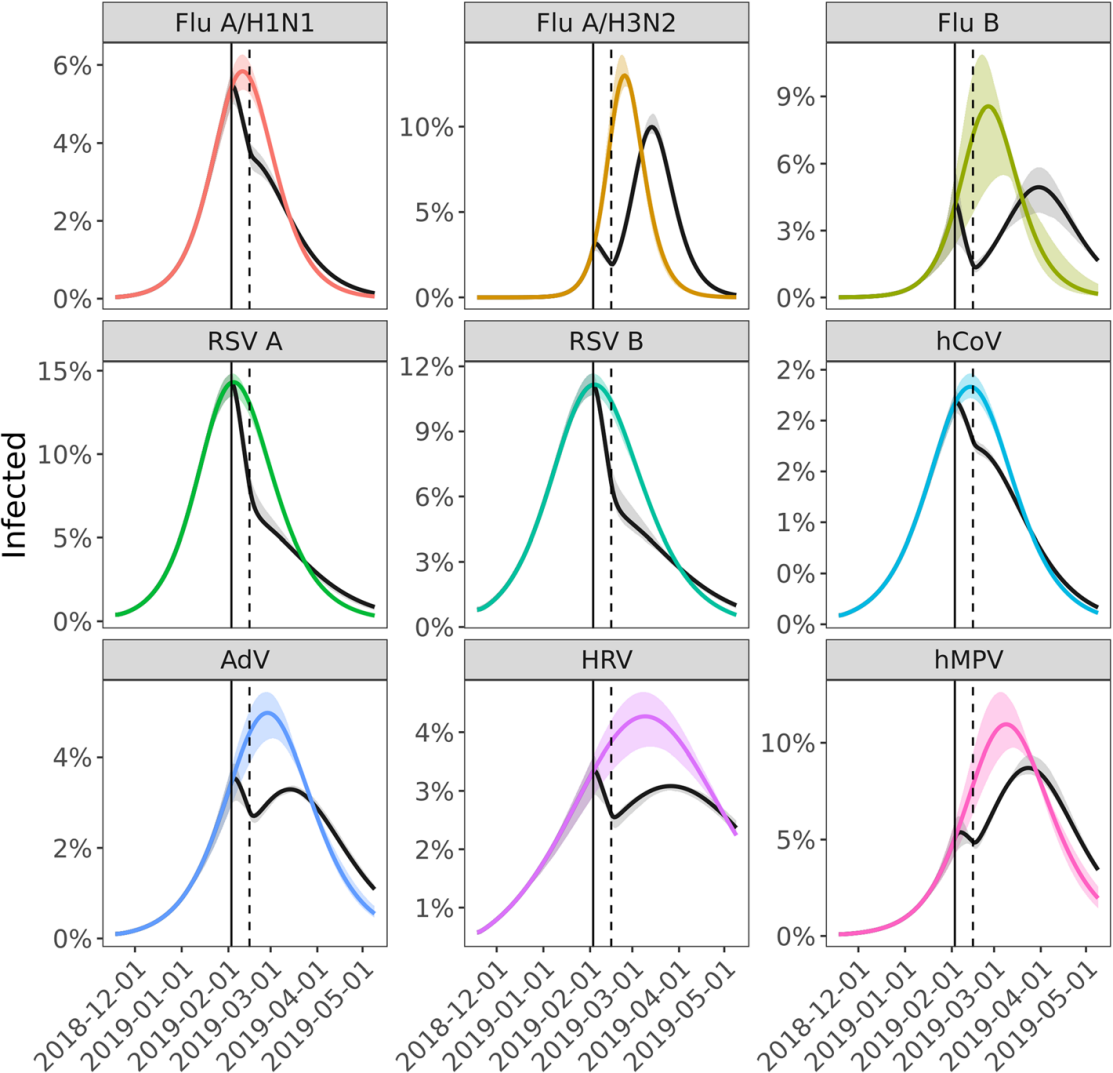
Effect of weather-related disruption on incidence. Percentage of the population infected over time for the best fit model (gray) and assuming no weather-related disruption in contact patterns (colored). The solid and dashed black lines mark the beginning and end of weather-related social distancing

We estimated the percent of infections averted by weather-related city-wide disruption by simulating transmission of each of these nine viruses with and without the presence of decreased effective contact rates and compared incidence of infection under both scenarios. The percent of infections averted ranged from 3.0% (95% CI, 2.0–3.7%) for human metapneumovirus to 9.2% (95% CI, 6.2–10.3%) for RSV B (Table 1). Comparing the pathogens with the highest incidence, influenza A/H1N1 and influenza A/H3N2, we observed that the weather-related disruption occurred shortly before the predicted peak of the influenza A/H1N1 epidemic but early in the course of the influenza A/H3N2 epidemic (Fig. 3). The estimated impact of the disruption was significantly greater for A/H1N1 (7.6% of infections averted, 95% CI, 5.2–8.7%) than for A/H3N2 (3.1% averted, 95% CI, 2.5–3.2%). For A/H1N1, the weather-related transmission disruption appears to have reduced incidence without significant rebound, while the main effect on A/H3N2 was to delay the peak in incidence by an estimated 18 days (95% CI, 17–21 days).

**Fig. 4:**
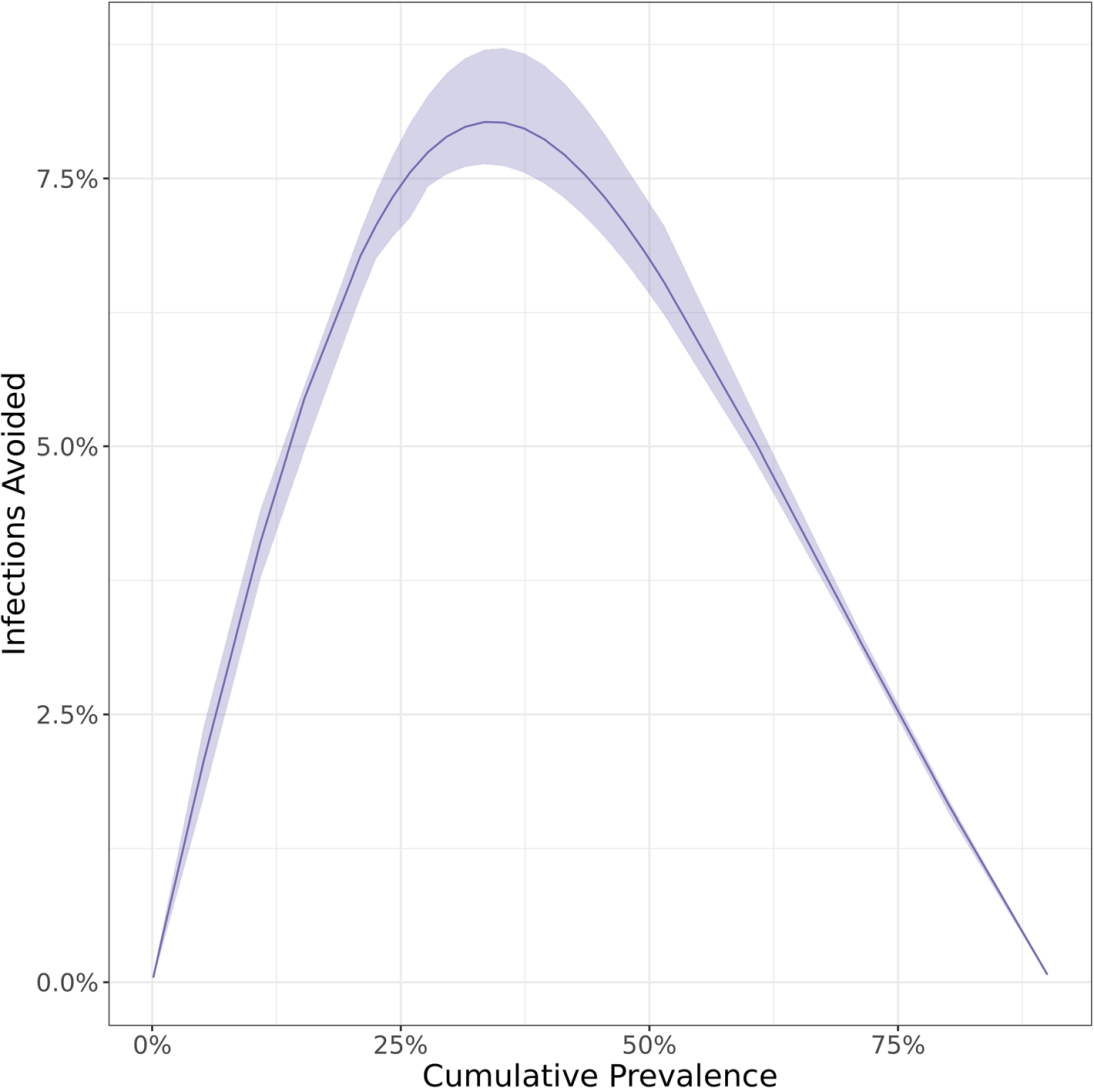
Estimated proportion of influenza A/H3N2 infections averted by a 14-day reduction in contact patterns analogous to those caused by the February 2019 extreme weather disruption, based on cumulative incidence at the time the contact reduction begins

To characterize the effect of the timing of social distancing on epidemic incidence, we simulated an unmitigated Flu A/H3N2 (most infectious of the 9 pathogens) epidemic, and then with a 14-day disruption starting at different points during the epidemic. Because time until the epidemic peak is likely unknown, we timed the interventions based on cumulative incidence. Early in the course of the epidemic, a 14-day disruption by itself, has little effect on the final epidemic size (Fig. 4). In contrast, later disruptions can meaningfully reduce peak incidence. For example, a disruption starting when cumulative incidence reaches 32% was predicted to reduce total incidence by 15.9%.

In supplemental analyses, we found that variation within reasonable ranges of the fixed parameters does not affect the percent of infections averted, with the exception of the length of latent and infectious periods. These showed trends to lower the infections averted as they got longer, but this effect was not significant except for Flu A/H3N2 and even there the effect was fairly small and does not qualitatively change the conclusions, such as what is shown in Fig. 4.

### Discussion

Modeling and simulation studies have been used to predict the impact of social distancing on the course of a hypothetical influenza pandemic (e.g. [28–31]). Although there is heterogeneity in the specific form of social distancing modeled, most of the studies have assumed that social distancing measures are initiated early in the course of a pandemic. These studies consistently suggest that such social distancing can delay the timing and intensity of the pandemic peak, but with little impact on the final attack rate of the pandemic. Our observations support these findings. The extreme snowfall of February 2019 occurred early in the seasonal epidemics of influenza A/H3N2 and hMPV, and we estimate that the final attack rates of these viruses only decreased by 3% and the peaks only shifted by 18 or 15 days, respectively. This has important implications for the current COVID-19 pandemic, where early control has generally suppressed transmission, and total release of control measures (without compensation from masks, vaccination, etc.) would be expected to lead to peaks nearly as intense as if no control measures had been attempted [32].

In contrast, the extreme snowfall occurred close to the predicted peak of the seasonal epidemics of several other viruses, particularly influenza A/H1N1 and RSV. For these viruses, weather-related social distancing had larger impacts, with final attack rates reduced by 7.6 to 9.2% and prevalence remaining below the pre-disruption level for the remainder of the season. This finding suggests that short-term social distancing is most effective at reducing overall attack rates when it occurs close to the peak of the epidemic, or equivalently when force of infection is greatest. There are important differences between the respiratory viruses we model and SARS-CoV-2, such as the burden and possible overwhelming of the healthcare system and the role of different age groups in spreading the virus. However, for localities that have lost control of the current pandemic, interventions can still reduce the expected burden of COVID-19. As the pandemic progresses even short measures can have significant effects.

It is unlikely that traffic and school closure data fully capture the changes in population contacts due to extreme snowfall. For this reason, we did not attempt to parse the relative impacts of specific changes on routes of transmission. However, pathogens that tended to infect younger age groups also tended to have greater reductions in effective contact rates. This suggests that weather-related school closures may have had a greater effect on contact frequencies among children than commuting and workplace disruptions among adults.

Several limitations of this study should be considered. First, the specimens from this study came from patients evaluated in hospitals. These were chosen to minimize changes in detection due to behavior (i.e. patients with mild illness may be less likely to seek care during the extreme weather). If respiratory virus illness in hospital settings does not fully represent underlying illness in the community, our results may be biased. Second, we assumed latent and infectious periods for each pathogen based on the existing literature, which is more comprehensive for some viruses (e.g. influenza) than for others, though we partly address this with the sensitivity analysis (see SI). Finally, our model is a simple mass-action model and does not attempt to account for potential heterogeneity in the population by age, risk status, or other factors.

### Conclusions

Simulation studies to estimate the impact of social distancing on an influenza pandemic [28–31] have generally assumed that social distancing is implemented early in the pandemic and maintained until the end of the pandemic, a period of many months. Here we show that short periods of social distancing can lead to important reductions in respiratory virus transmission, even after control has been lost.

## Supplementary Information

**Additional file 1.**

## Data Availability

The datasets and code used during the current study are available from the corresponding author on reasonable request.

## Abbreviations

RSV: Respiratory syncytial virus
hCoV: Human coronavirus
hMPV: Human metapneumovirus
HRV: Rhinovirus
AdV: Adenovirus
SEIR: Susceptible-Exposed-Infectious-Recovered
