# Supplementary material for: Effects of weather-related social distancing on city-scale transmission of respiratory viruses: a retrospective cohort study
Source: BMC Infect Dis. 2021 Apr 9;21:335. doi: 10.1186/s12879-021-06028-4 (PMC8033554; doi:10.1186/s12879-021-06028-4)
Supplement: Supplementary file 1 — Additional file 1. [file 12879_2021_6028_MOESM1_ESM.docx]

**Supplemental Model Information**

As mentioned in the main text we model each pathogen with following SEIR model:

$dS/dt = -\beta(1 +\beta^{'}\theta_{t})SI$

$dE/dt = \beta(1 +\beta^{'}\theta_{t})SI - \sigma E$

$dI/dt =\sigma E -\gamma I$

$dH/dt =p(1 +\alpha\theta_{t})(dI/dt) +p\alpha(\delta t_{start}-\delta t{}_{end})I$

There are three types of parameters in this model. First are the shared fixed parameters ( t_start_ =3 Feb, t_end_=15 Feb, and *α*=-10%). Here and in the main text we talk about the duration of the disruptions (t_end_-t_start_) instead of t_start_ and t_end_; this duration is adjusted by changing t_end_. All three of these parameters are defined based on the data in Figure 1. Next we have the individual fixed parameters ($\sigma$ and $\gamma$) which are set based on literature (see Table 1). Lastly, we have the individually estimated parameters (*β*, *β*’, *p*, $I_{0}$). Of these parameters, *β*’ (strength of the disruption in transmission) is the one of greatest interest. Inferring this value from data instead of exploring what theoretical levels of social distancing can do is the heart of this paper.

We fit the model using the Poisson (log) likelihood:

$-logL=\sum_{t=1}^{N} (-{H_{t}}^{obs}log{H_{t}}^{model}+{H_{t}}^{model})$

The model and all analysis were done in R (version 3.6.2). The model was implemented in compiled C using the Csnippet function of the pomp library (version 2.2). Point estimates of the maximum likelihood and profiles over specific variables were done with optim and GenSA (version 1.1.7). All evaluations of the model were saved to build up the posterior distribution, used for the confidence intervals (described in the main text).

We ran sensitivity analyses on the four fixed parameters (length of disruption, *α*, $\sigma,$ and $\gamma$). For each variable we choose five different values; for each value we refit the model and calculated the percent of infections averted.

We varied the length of disruption from 6 to 14 days. Figure S1 shows that there is little dependence on the length of disruption for the range we tested, except for RSV A and B, which at shorter durations show marked drops in the infections averted. This is not unexpected because of the longer latent and infectious period of RSV. Furthermore, this drop is not significant until we get to 8 days (t_end_=11 Feb) which would put the end of the disruption in the middle of the second drop in traffic (see Figure 1) making it unlikely to be a realistic duration.

We varied *α* from 0% to -20% (twice the inferred value, see Figure 1). Figure S2 shows no significant effect on the infections averted under these circumstances.

For $\gamma$ we varied the duration of infectiousness for -2 to +2 days for the value used in the main text. We see (unsurprisingly) a trend to lower the infections averted as the infectious period gets longer. However, this trend is not more significant than the uncertainty in the infections averted, with the notable exception of Flu A/H3N2. Flu A/H3N2 has very little uncertainty making the change in infections averted significant. Even with this, the qualitative conclusions are the same: intervening early but failing to control (A/H3N2) gives less protection than that same intervention late (A/H1N1).

The story for $\sigma$is much the same as for $\gamma$. We varied the latent period from -2 to +2 days of the value used in the main text. However, for influenza and rhinovirus we discarded the -2 value (period = 0, $\sigma$=infinity) for numeric reasons. There is a trend to lower infections averted with a longer period but it is not significant except perhaps for Flu A/H3N2.


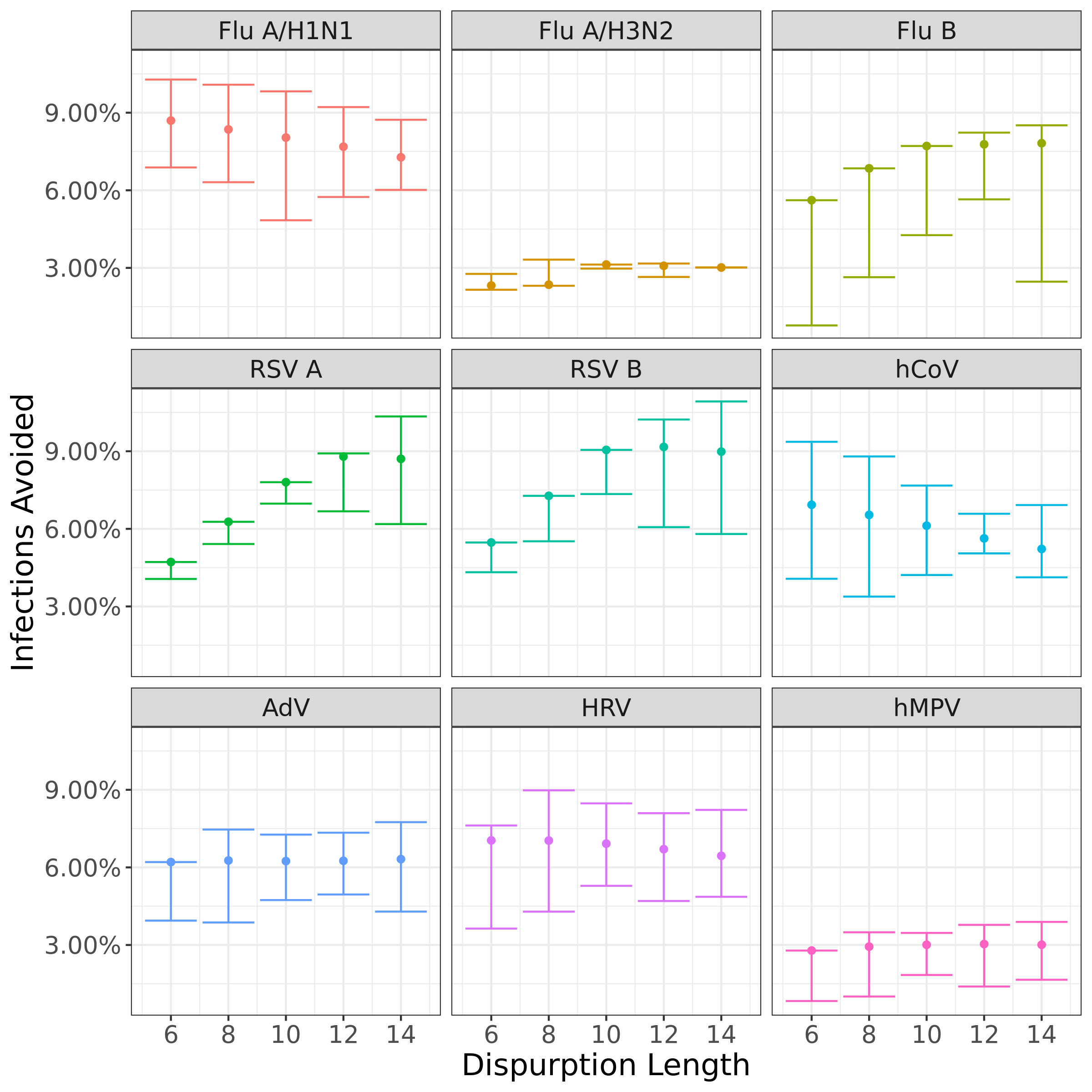


**Figure S1:** Infections averted across 9 pathogens as we vary the assumed length of the mobility disruption


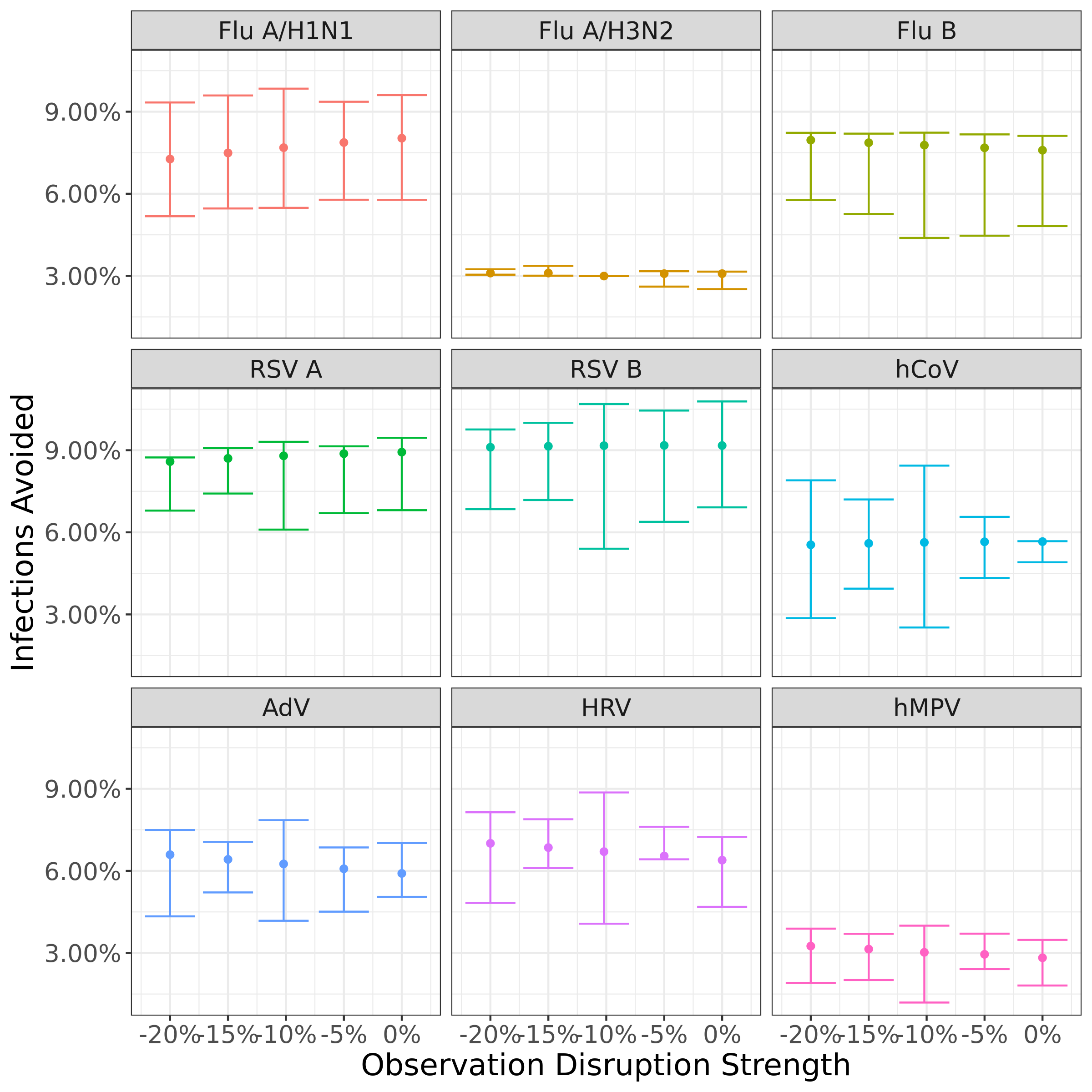


**Figure S2:** Infections averted across 9 pathogens as we vary the assumed change in observation disruption.


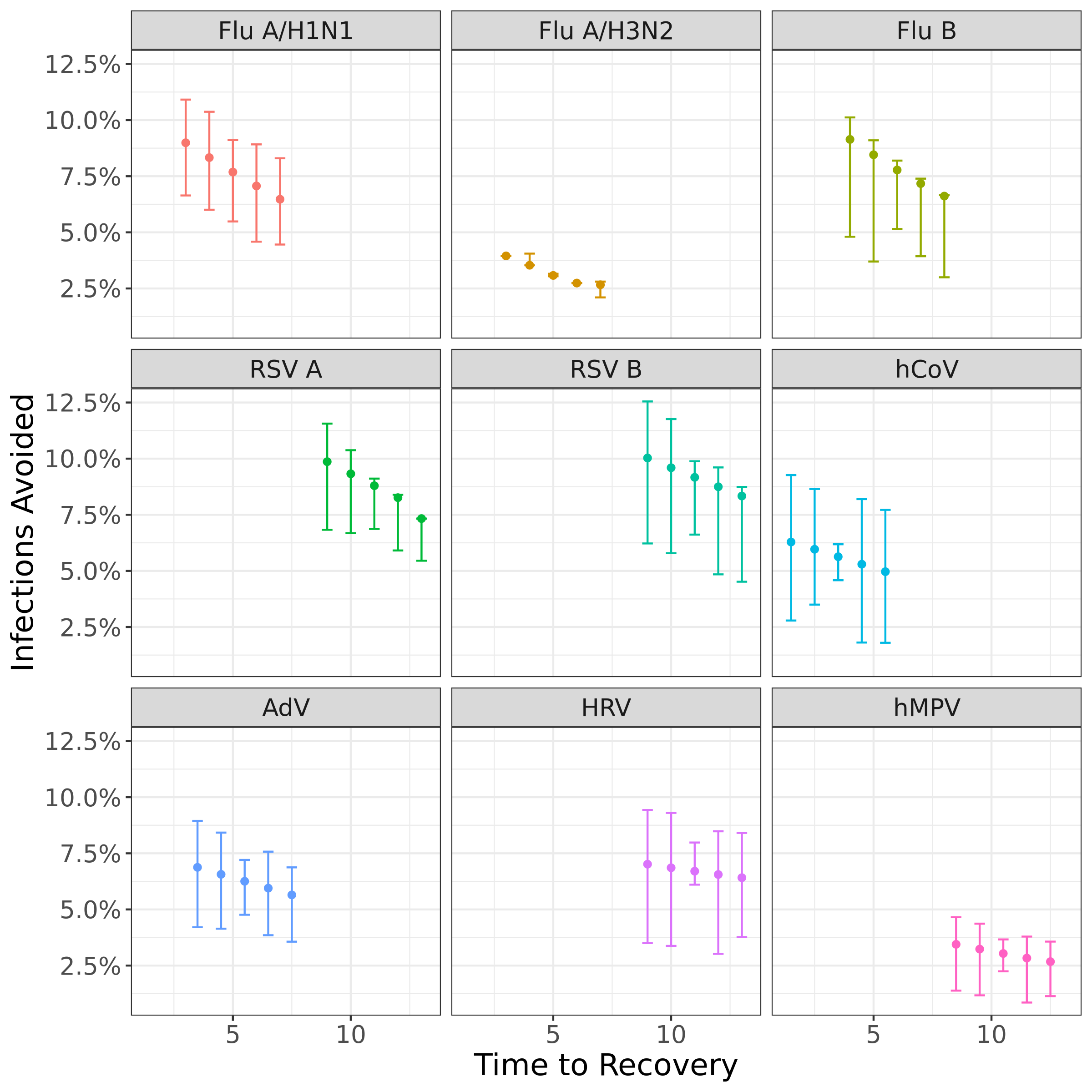


**Figure S3:** Infections averted across 9 pathogens as we vary the assumed time to recovery (1/$\gamma$).


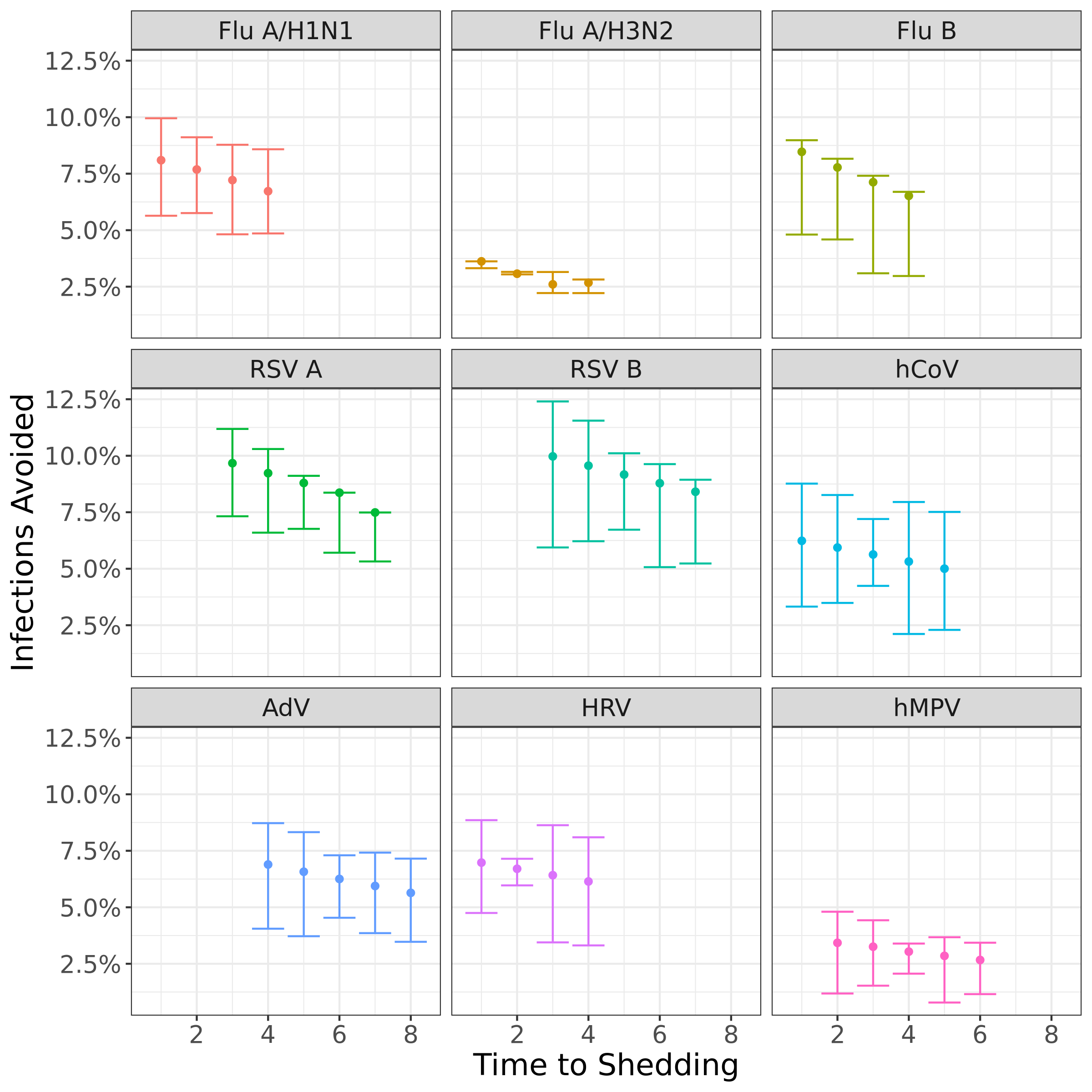


**Figure S4:** Infections averted across 9 pathogens as we vary the assumed time to shedding (1/$\sigma$).

**Supplemental Table: Participant Demographics (available for 5,691 of 7,555 unique patients)**

|  | **N=5691**  *N (%) unless otherwise specified* |
| --- | --- |
| ***Age*** |  |
| Median [IQR] | 6 [2, 25] |
| < 18 years old | 4099 (72.0) |
| **Male Sex** | 3160 (55.5) |
| **Race** |  |
| White | 2647 (46.5) |
| Black/African American | 737 (13.0) |
| Asian | 631 (11.1) |
| American Indian/Alaska Native | 73 (1.3) |
| Native Hawaiian/Pacific Islander | 102 (1.8) |
| Other Race | 900 (15.8) |
| Multiple Races Listed | 267 (4.7) |
| *Missing* | *334 (5.9)* |
| **Hispanic Ethnicity** | 1216 (21.4) |
| *Missing* | *214 (3.8)* |
| **Insurance Status** |  |
| Private | 1891 (33.2) |
| Government | 3032 (53.3) |
| Other | 155 (2.7) |
| *Missing* | *613 (10.8)* |
